# Longitudinal Changes in Physical Function and Their Impact on Health Outcomes in COVID-19 Patients

**DOI:** 10.3390/nu15204474

**Published:** 2023-10-22

**Authors:** Rebecca De Lorenzo, Luigi Di Filippo, Sabrina Scelfo, Aurora Merolla, Andrea Giustina, Caterina Conte, Patrizia Rovere-Querini

**Affiliations:** 1 School of Medicine and Surgery, Vita-Salute San Raffaele University, 20132 Milan, Italy; delorenzo.rebecca@hsr.it (R.D.L.); difilippo.luigi@hsr.it (L.D.F.); merolla.aurora@hsr.it (A.M.); giustina.andrea@hsr.it (A.G.); rovere.patrizia@hsr.it (P.R.-Q.); 2 Institute of Endocrine and Metabolic Sciences, Vita-Salute San Raffaele University, IRCCS Hospital, 20132 Milan, Italy; 3 Division of Immunology, Transplantation, and Infectious Diseases, IRCCS San Raffaele Scientific Institute, 20132 Milan, Italy; sabrinascelfo@alice.it; 4 Department of Human Sciences and Promotion of the Quality of Life, San Raffaele Roma Open University, 00166 Rome, Italy; 5 Department of Endocrinology, Nutrition and Metabolic Diseases, IRCCS MultiMedica, 20099 Milan, Italy; 6 Innate Immunity and Tissue Remodeling Unit, Division of Immunology, Transplantation, and Infectious Diseases, IRCCS San Raffaele Scientific Institute, 20132 Milan, Italy

**Keywords:** SARS-CoV-2, COVID-19, muscle strength, sarcopenia, physical function

## Abstract

Background: Coronavirus disease (COVID-19) is correlated with a variety of long-term sequelae that affect different aspects of health, including physical function. This study investigated the longitudinal changes in handgrip strength (HGS) over six months post-hospital discharge in COVID-19 patients and explores the associations between HGS, health-related quality of life, dyspnoea, exercise capacity, and body mass index (BMI). Methods: Adult COVID-19 patients were followed up at one, three, and six months after hospital discharge. HGS, BMI, exercise capacity, and health-related quality of life were assessed. Data from patients with HGS measurements at all three time points were analysed. Results: Low HGS was prevalent one month post-discharge (35%). Participants with low HGS exhibited more severe disease (30.5% vs. 5.9% were admitted to the intensive care unit, *p* < 0.01), longer hospital stays (median [IQR] 21 [10.0; 40.5] vs. 12.0 [8.0; 20.0] days, *p* < 0.01), greater weight loss (−5.7 [−9.1; −0.6] vs. −3.2 [−5.7; −0.0] kg, *p* = 0.004), and reduced exercise capacity (6 min walking test [6 MWT], 95.7 [84.0; 102.0] vs. 100.0 [92.9; 105.0]% predicted, *p* = 0.007). Those with persistently low HGS (40% of the initial low HGS group) had worse exercise capacity (6-MWT 93.3 [78.3; 101.0] vs. 101.0 [95.0; 107.0]% predicted, *p* < 0.001), more dyspnoea (29.0% vs. 2.0% of participants, *p* < 0.001), poorer quality of life (visual analogue scale score, 75 [50; 75] vs. 85 [75; 95], *p* < 0.001), and higher rates of problems in various health dimensions. HGS at 1 month was the only significant predictor of HGS improvement from 1 month to 6 months (odds ratio [95% CI] 1.11 [1.03; 1.20], *p* = 0.008). Conclusions: This study highlights the prevalence of reduced physical function among COVID-19 survivors and emphasises the importance of early identification and intervention to optimise their long-term health. Monitoring HGS, a simple and reliable tool, can provide valuable insights into patients’ overall physical function, aiding in tailored care and improved outcomes.

## 1. Introduction

COVID-19 has been linked with long-term effects, including respiratory, neurological, metabolic, endocrine, and musculoskeletal alterations [1,2] that deeply affect the quality of life [3]. These signs and symptoms comprise complex and varied scenarios identified as long-COVID or post-COVID syndrome [2]. Up to 50% of individuals with COVID-19 have been reported to have long-COVID [4,5], the most common symptoms being fatigue and dyspnoea [6]. Long-COVID is more common in those with severe illness but can affect anyone exposed to SARS-CoV-2 [7]. The main risk factors include older age and female sex, in addition to severe illness. The risk is further increased by comorbid conditions such as diabetes, obesity, and respiratory diseases [8,9,10]. Reports of unintentional weight loss and malnutrition have been frequent among COVID-19 patients, independent of disease severity [11,12,13,14,15]. Loss of lean body mass caused by systemic inflammation, anorexia, taste loss, muscle disuse, and bed rest contribute to the weight loss associated with COVID-19 [16]. Although most patients regain weight after the acute phase, this appears to be due to a disproportional increase in adiposity, which may further worsen their body composition [17]. Based on this background, COVID-19 bears a high risk of sarcopenia, a reduction in muscle mass and strength [18]. Consistently, several authors have reported that COVID-19 survivors have reduced physical performance, independent of disease severity [19,20,21,22,23]. Handgrip strength (HGS) is the force generated by the hand muscles when gripping an object. It is a simple and inexpensive measure of muscle strength and function that is measured using a handgrip dynamometer (hydraulic, pneumatic, or mechanical), and is associated with a variety of health outcomes [24]. HGS also reflects several health outcomes, including health status, mortality risk, and length of hospital stay [25,26], and is a key criterion for the diagnosis of sarcopenia, a condition characterised by low muscle mass and strength [27]. SARS-CoV-2 infection increases the risk of clinically relevant reductions in HGS [28]. Low muscle strength, as measured using the HGS test, was present in 65% of men and 47% of women transferred to a pulmonary rehabilitation facility after hospitalisation for COVID-19 [29]. HGS was lower in malnourished individuals and was correlated with malnutrition scores [29]. Currently, there is a limited understanding of the dynamics of physical function and their influence on the health outcomes of COVID-19 patients in the months after hospital discharge. It has been reported that low HGS is present in 52% of COVID-19 patients 3–11 months after discharge from hospital [30]. However, that investigation was cross-sectional, and data from individuals assessed at different times were presented in an aggregate form, not allowing a characterisation of changes in physical function over time.

With the belief that COVID-19 might serve as a multiscale modelling framework to study the effects of reduced physical function on health outcomes beyond COVID-19 itself, we sought to evaluate the longitudinal changes in physical function measured using the HGS test over 6 months after hospital discharge in COVID-19 patients, and changes in health-related quality of life, dyspnoea, exercise capacity, and body mass index (BMI) in relation to physical function. The factors associated with an improvement in HGS were also investigated. We hypothesised that impaired physical function soon after COVID-19 significantly impacts long-term health-related quality of life and overall health status.

## 2. Materials and Methods

### 2.1. Study Design

This was a sub-study of the COVID-BioB study, a large prospective observational investigation performed at San Raffaele University Hospital, a tertiary healthcare hospital in Milan, Italy [31,32]. The study protocol complied with the Declaration of Helsinki, was approved by the Hospital Ethics Committee (protocol no. 34/int/2020), and was registered on ClinicalTrials.gov (NCT04318366). Full descriptions of patient management and clinical protocols have been previously published [31,32]. Signed informed consent was obtained from all the patients participating in this study. We included adult (age ≥ 18 years) individuals with a confirmed diagnosis of COVID-19 who had been admitted to and subsequently discharged home from a COVID-19 medical ward of San Raffaele University Hospital, and were re-evaluated one, three, and six months after remission at the Outpatient COVID-19 Follow-Up Clinic of the same institution. All outpatients who completed all follow-up visits (1, 3, and 6 month) between 30 November 2020 and 18 November 2021 were included. Study size was defined by the time window of the study. Confirmed COVID-19 was defined as positive real-time reverse-transcriptase polymerase chain reaction (RT-PCR) from a nasal and/or throat swab, together with signs, symptoms, and/or radiological findings suggestive of COVID-19 pneumonia. Remission was defined as two negative RT-PCR results from a nasal and/or throat swab performed 24 h apart with no symptoms. Only patients with available anthropometric data (weight and height) recorded upon admission and at follow-up and HGS tests at 1-month, 3-month and 6-month visits were included in the analyses. Patients who were admitted for other reasons and subsequently diagnosed with superimposed SARS-CoV-2 infection were excluded.

### 2.2. Data Collection

The Outpatient COVID-19 Follow-up Clinic is staffed by a multidisciplinary team encompassing specialists in internal medicine, neurology, psychiatry, cardiology, nutrition, and nephrology. This team conducted a retrospective analysis of patient medical records, evaluating both the initial presentation of COVID-19 and the subsequent disease progression. These assessments occurred in the presence of the patient and were meticulously integrated with their comprehensive medical history. Moreover, a comprehensive physical examination was carried out. Data were entered into a dedicated electronic case record form (eCRF) specifically developed for the COVID-BioB study. Prior to the analysis, the data were cross-checked with medical charts and verified by data managers and clinicians for accuracy. The following variables were collected for all participants: age, sex, race/ethnicity, BMI, comorbidities (including history of arterial hypertension, diabetes mellitus, chronic kidney disease, ischaemic heart disease, and active malignancy), length of stay (LoS), and therapeutic regimen (low-flow oxygen, non-invasive mechanical ventilation [NIV], or admission to the Intensive Care Unit [ICU]). Measuring weight and height on admission was not feasible due to the workload of nurses and physicians during the peak of the pandemic and the need for contact and airborne precautions in the hospital. Therefore, the weight and height on admission were self-reported by the patients. Height measured at follow-up visits was subsequently used to calculate baseline BMI for the present analysis.

The first follow-up outpatient visit was scheduled one month after discharge (1 M), the second visit at three months (3 M), and the third visit at six months (6 M). All visits included a complete internal medicine assessment (collection of medical history, measurement of vital signs, physical examination) and nutritional evaluation (body weight, height, waist circumference measurements, nutritional questionnaires, and muscle function and strength evaluations). Body weight was measured to the nearest 0.1 kg using a balance-beam scale, height was measured to the nearest 0.1 cm using a wall-mounted stadiometer, and BMI was calculated as the ratio between the weight (kg) and the height (m) squared. Participants were classified into four groups according to their BMI: underweight (BMI < 18.5 kg/m^2^), normal weight (BMI 18.5–24.9 kg/m^2^), overweight (BMI 25.0–29.9 kg/m^2^), and obese (BMI ≥ 30 kg/m^2^). Waist circumference measurements were performed around the abdomen at the level of the umbilicus. Abdominal obesity was defined as waist circumference ≥88 cm in women and ≥102 cm in men.

Nutritional status was evaluated using the Mini Nutritional Assessment Short Form (MNA-SF) and classified according to the resulting score as follows: normal nutritional status, MNA-SF score ranging from 12 to 14; risk of malnutrition, MNA-SF score ranging from 8 to 11; and malnutrition, MNA-SF score ranging from 0 to 7 [33].

Physical function was assessed using the HGS test with a digital dynamometer (Jamar Plus; Paterson Medical, Green Bay, WI, USA). The mean value of three consecutive measurements performed using the dominant hand was used. Participants with HGS values below the 5th percentile for sex and age were classified as having low muscle strength (low HGS) [34]. Those whose HGS normalised (from below to above the 5th percentile) at 6 M were considered to have improved HGS.

Exercise capacity was evaluated using the 6 min walking test (6 MWT) as per the guidelines provided by the American Thoracic Society (ATS) [35]. A validated reference equation, developed in healthy subjects from seven different countries, was used to derive predictive values of the 6 min walk distance (6 MWD) in patients with no history of chronic pulmonary disease [36]. A different equation specific for chronic obstructive pulmonary disease (COPD) patients was used to calculate COPD-predicted values of the 6 MWD [37].

Health-related quality of life was assessed using the European Quality of Life Questionnaire’s three-level version (EQ-5D-3L) [38]. The EQ-5D-3L includes five dimensions (mobility, self-care, usual activities, pain/discomfort, and anxiety/depression) as well as a visual analogue scale (EQ VAS). Each dimension has three levels: no problems, some problems, and extreme problems. For the purposes of this analysis, EQ-5D levels were categorised as either ‘no problems’ (level 1) or ‘any problems’ (levels 2 and 3). The EQ VAS assesses the patient’s self-rated health on a vertical scale, with endpoints labelled as ‘best imaginable health state’ and ‘worst imaginable health state’.

### 2.3. Statistical Analyses

Descriptive statistics were obtained for all the study variables. Continuous variables are expressed as medians [25th–75th percentiles]. Categorical variables are summarised as counts and percentages. Fisher’s exact test or χ^2^ test and the Wilcoxon signed-rank test or the Kruskal–Wallis test were employed to assess differences in categorical and continuous variables, respectively. Correlations were analysed using the Spearman’s rank correlation analysis. Univariate and multivariate logistic regression analyses were used to identify the variables associated with HGS improvement from 1 M to 6 M. The demographic and clinical characteristics potentially associated with HGS improvement were tested using univariable models. All variables that emerged as predictors (*p* < 0.05) in the univariate analysis were used as covariates in the multivariate model. The missing data were not imputed. All statistical tests were two-sided. Statistical significance was set at *p* value < 0.05. Statistical analyses were conducted using IBM SPSS Statistics (IBM SPSS Statistics for Windows, Version 22.0. Armonk, NY, USA: IBM Corp.).

## 3. Results

### 3.1. Participant Characteristics upon Admission and Disease Severity

In total, 234 participants were included in the analysis. Their characteristics are summarised in Table 1. Most participants were male (61.5%), the median age was 67 years, and most (65%) were older than 60 years. All patients were hospitalised with a median LoS of 14 days. Most participants received low-flow oxygen or non-invasive mechanical ventilation (NIV), and 14.5% were admitted to the ICU (Table 1). The median BMI upon hospital admission was 28.2 kg/m^2^. The majority (79%) of participants were overweight (43.7%) or obese (35.5%), while 20.3% were normal weight, and 0.5% were underweight.

### 3.2. Anthropometrics and Physical Evaluations during Follow-Up

**Figure 1:**
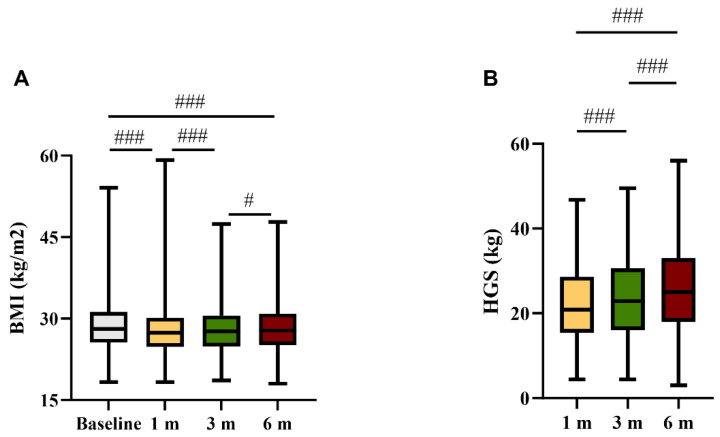
(**A**) Body mass index (BMI, kg/m^2^) prior to hospital admission and at 1, 3, and 6 months; and (**B**) handgrip strength (kg) at 1, 3, and 6 months after discharge. # *p* < 0.05; ### *p* < 0.01 within the same group.

The median BMI significantly decreased from baseline to 1 M, and progressively increased from 1 M to 6 M (Figure 1A). However, at 6 M the BMI was still significantly lower than that at baseline (Figure 1A). The median HGS progressively increased during the follow-up period (Figure 1B). At 1 M, 82 participants (35%) were classified as having low HGS (HGS < 5th percentile of reference values for age and sex).

### 3.3. Comparison between Participants with Normal and Low HGS

Table 2 presents a comparison between individuals with normal and low HGS. Participants with low HGS were younger and more frequently female than those with normal HGS (Table 2). The BMI and prevalence of comorbidities at hospital admission were similar between groups. Participants with normal HGS were treated more frequently with low-flow oxygen during hospitalisation, less commonly admitted to the ICU, and had a shorter LoS than those with low HGS (Table 2).

At 1 M, weight loss from the baseline was greater, and the proportion of participants with abdominal obesity was lower in those with low HGS. The percent predicted 6MWT was also lower in these participants compared to those with normal HGS (Table 2). At 3 M and 6 M, between-group differences in HGS and percent predicted 6MWT persisted (Table 2).

**Figure 2:**
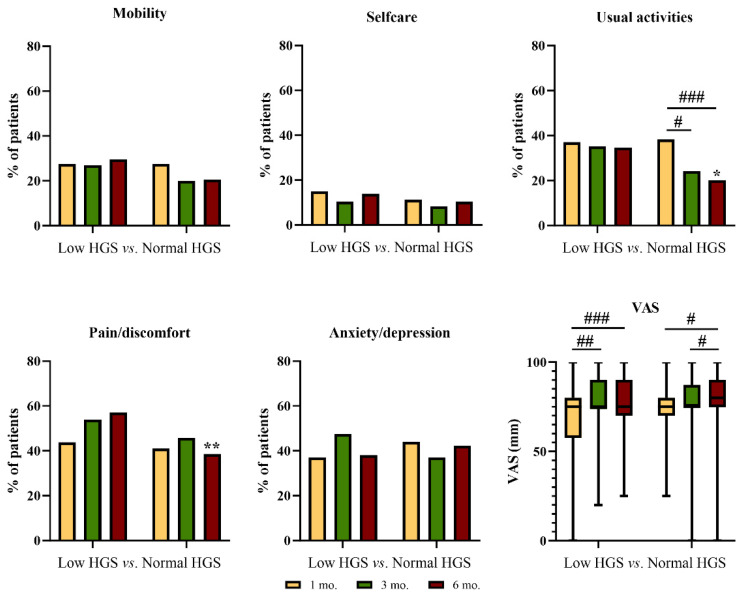
Problems in the EQ-5D dimensions and visual analogue scale (VAS) score reported at 1, 3, and 6 months after discharge by patients with low or normal handgrip strength at 1 month after discharge. # *p* < 0.05; ## *p* < 0.01, ### *p* < 0.01 within the same group * *p* < 0.05; ** *p* < 0.01 between groups.

In those with normal HGS at 1 M, the proportion of participants with at least some problems in performing usual activities significantly declined during follow-up, being significantly lower at 6 M as compared to the group with low HGS (Figure 2). At 6 M, the proportion of participants with moderate or extreme pain/discomfort was significantly lower among those with normal HGS than among those with low HGS (Figure 2). The general health status significantly improved from 1 M to 6 M in both groups (Figure 2).

### 3.4. Comparison between Participants with and without HGS Improvement

More than half (62%) of the participants with low HGS at 1 M had an improvement in HGS at 6 M, whereas HGS remained stable or worsened in the remainder.

The between-group differences are summarised in Table 3. Age, sex, baseline BMI, comorbidities, in-hospital treatments, and LoS were similar between groups (Table 3).

Participants with a stable or worse HGS had a significantly lower percent predicted 6MWT at all time points than those with an improved HGS (Table 3). Moreover, dyspnoea was more common among participants with a stable or worse HGS at 3 M and 6 M (Table 3).

**Figure 3:**
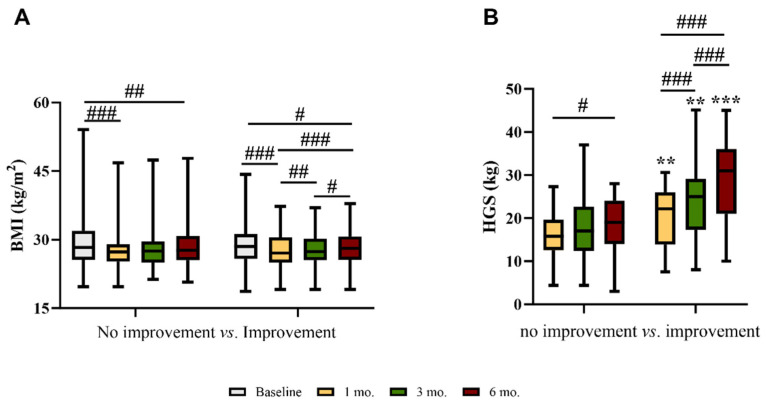
(**A**) Body mass index (BMI) and (**B**) handgrip strength (HGS) at baseline, 1, 3, and 6 months after discharge in patients with low HGS at 1 month whose strength did or did not improve at 6 months after discharge. # *p* < 0.05; ## *p* < 0.01; ### *p* < 0.001 within the same group ** *p* < 0.01; *** *p* < 0.001 between groups.

At 6 M, the median BMI was still significantly lower than that at baseline in both groups (Figure 3A). However, in those with an improvement in HGS, the median BMI progressively increased from 1 M to 6 M (Figure 3A).

As expected, in participants with an improvement in HGS, strength progressively increased from 1 M to 6 M (Figure 3B). There was a significant, although weak, correlation between changes from 1 M to 6 M in HGS and BMI (r = 0.187; *p* = 0.004).

**Figure 4:**
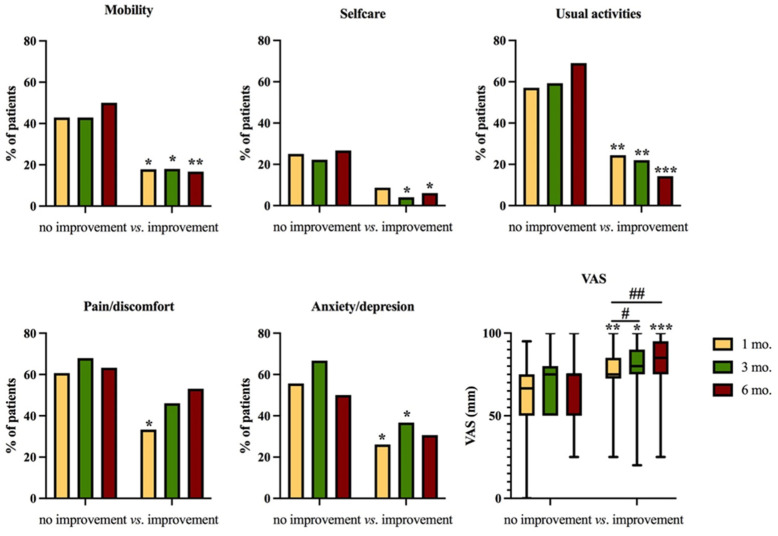
Problems in the EQ-5D dimensions and visual analogue scale (VAS) score reported at 1, 3, and 6 months after discharge in patients with low handgrip strength at 1 month whose strength did or did not improve 6 months after discharge. # *p* < 0.05; ## *p* < 0.01 within the same group * *p* < 0.05; ** *p* < 0.01; *** *p* < 0.001 between groups.

Finally, problems in the EQ-5D dimensions were significantly less common among those with an improvement in HGS (Figure 4), who also had a progressive improvement in general health status (VAS, Figure 4).

### 3.5. Predictors of HGS Improvement

In univariable logistic regression, HGS at 1 M and race/ethnicity emerged as significant predictors of HGS improvement from 1 M to 6 M. Only HGS at 1 M remained significant in the multivariable regression analysis (Table 4).

## 4. Discussion

This is the first study that assessed the longitudinal changes in physical function measured using the HGS test over 6 months following hospital discharge in COVID-19 patients, and the relationship between these changes and variations in health-related quality of life, dyspnoea, exercise capacity, and BMI. Low physical function was prevalent in COVID-19 survivors one month after hospital discharge, with 35% of the cohort having HGS values below the 5th percentile of the reference values for age and sex. As expected, participants with low HGS had more severe disease and a longer LoS. One month after discharge, the participants with low HGS exhibited greater weight loss from baseline and a worse exercise capacity (6 MWT). Although at six months the differences in HGS values between participants with low and those with normal HGS at one month were markedly reduced, the exercise capacity in the first group remained significantly lower. Furthermore, at all follow-up visits, participants whose HGS had not improved (40% of those with low HGS at 1 M) had a worse exercise capacity and higher rates of dyspnoea than those whose HGS had improved. Furthermore, at the six-month mark, these participants experienced more issues in EQ-5D dimensions and had lower VAS scores, indicating a strict correlation between physical function, as evaluated using the HGS test, and quality of life.

Several previous studies have reported impaired physical function in COVID-19 survivors. Reduced 6 MWD (<80% of the predicted value) was reported in 48% of 46 patients treated with mechanical ventilation during the acute phase and evaluated at 3 months following hospital discharge [19]. Similar proportions of patients with abnormal exercise capacity have been described in other reports [20,21,22,23,39], revealing that this is a prevalent and relevant issue among individuals who have recovered from COVID-19, and highlighting the need for early identification of these patients to facilitate tailored interventions and optimise their overall care. In our cohort, HGS was the only significant predictor of improved physical function (HGS) at six months, and people with low HGS had a worse exercise capacity, dyspnoea, and quality of life. Our findings are consistent with a large Canadian study that identified fatigue as a significant predictor of declining health-related quality of life after COVID-19 [40]. Although physical function and quality of life improve over time in COVID-19 survivors, an impairment in these health-related outcomes persists in a significant proportion of patients [40,41]. These data suggest that HGS should be assessed soon after discharge to identify patients who may need additional care to improve their health status.

Assessing HGS is a straightforward and cost-effective approach for evaluating muscle strength and function, and correlates with strength in other body parts, making it a dependable substitute for more complex measures of lower and upper extremity strength [27]. Accurate measurement of HGS requires the use of a calibrated handheld dynamometer in well-defined test conditions with normative data from suitable reference populations.

HGS is a strong predictor of poor long-term clinical outcomes. In a prospective study conducted in 17 different countries, including 142,861 participants followed for 4 years from 2003 to 2009, the HGS values were inversely correlated with all-cause mortality, cardiovascular mortality, and ischaemic, cardiovascular, and neurological events. Moreover, HGS was found to more effectively predict all-cause and cardiovascular mortality compared to systolic blood pressure [42]. Other studies have shown a strict inverse association between HGS levels and the risk of cardiovascular disease, diabetes, traumatic falls and fractures, osteoporotic complications, major depression, and cognitive deficits occurrence [43,44,45,46,47,48]. A systematic review and meta-analysis including 504 studies and 8 systematic reviews that evaluated the influence of HGS on 11 different adverse clinical outcomes reported a strong negative influence of low HGS, particularly on all-cause and cardiovascular mortality and disability [26]. The authors concluded that HGS might represent an extremely reliable and easy-to-use clinical tool to assess participants’ general health status and risk of adverse events.

Our data confirmed our previous finding that unintentional weight loss is frequently observed in COVID-19 survivors [13]. Although most participants return to their original weight after recovery [17], here we have shown that six months after discharge, the BMI is still lower than baseline, and that weight gain is associated with HGS improvement. It has been estimated that the prevalence of malnutrition among individuals hospitalised with COVID-19 ranges between 12% and 83% in general ward patients and 31% and 94% in patients admitted to the ICU [49]. An association between malnutrition and HGS has been reported in COVID-19 patients [29], prompting the need for nutritional interventions aimed at improving physical function in this population. Several nutritional recommendations have been published for the management of individuals with COVID-19 [50,51]. It is generally acknowledged that the risk of malnutrition should be evaluated in vulnerable populations and in patients who are hospitalised. Specific guidelines for energy, protein, and fluid intake are provided, and oral nutritional supplements may be recommended if a patient’s nutritional requirements are not being met [51]. However, a systematic review and meta-analysis has shown that nutritional interventions, such as oral nutritional supplements, dietary counselling, or a combination of both, do not lead to improvements in HGS in older adults who are malnourished or at risk of malnutrition. [52]. More research is needed to verify the efficacy of the proposed nutritional interventions on HGS and other clinically relevant outcomes in people who survive COVID-19. In contrast, multidimensional respiratory rehabilitation programs incorporating respiratory physiotherapy, and aerobic, strength, and resistance training result in gradual recovery of functional capacity, leading to increased autonomy and improved quality of life [53,54]. A large meta-analysis found that rehabilitation interventions demonstrated an association with improved outcomes in functional exercise capacity, dyspnoea, and quality of life [55]. In this light, we propose that a physical therapy professional should ideally be included in the management of patients admitted to medicine wards. Physical therapists help patients regain mobility, manage pain, and improve their quality of life. This tailored care approach accelerates recovery, reduces complications and readmissions, and empowers patients to actively participate in their health [56,57].

Notably, while the proportion of participants with abdominal obesity was significantly lower among those with low HGS at 1 M, this figure progressively grew over time, reaching a proportion similar to that of people with normal HGS at 6 M (Table 2). Among those with low HGS, the proportion of individuals with abdominal obesity was numerically greater in the subgroup with no improvement in HGS at any time point (Table 3). These findings suggest unfavourable alterations in body composition, including a progressive increase in abdominal adiposity over time. In light of these observations, the importance of nutritional counselling and physical rehabilitation becomes even more evident.

This study has limitations, so the data should be interpreted with caution. First, its retrospective design hampers the generalisability of the findings. Weighing patients during their hospital stay was unfeasible due to the unprecedented workload of healthcare professionals in the first wave of the pandemic. This might have led to only partially accurate weight values. Data on body composition were not available, which did not allow us to identify the participants as sarcopenic. A diagnosis of sarcopenia requires both muscle mass and function criteria [27]. Finally, we did not have data on physical performance and HGS before hospitalisation, which might have been useful for investigating the influence of pre-disease muscle function on acute and long-term outcomes. A strength of our study is the inclusion of a large sample of participants who were hospitalised with COVID-19 during the first wave of the pandemic, reducing the potential bias due to anti-SARS-CoV-2 vaccination. Moreover, its longitudinal nature with patient assessments at different time points after discharge allowed us to evaluate the dynamics of the study outcomes and determine whether physical function trajectories, rather than single punctual values, impact long-term health status and quality of life. This brings a novel perspective to the field of post-COVID-19 health status. By establishing a clear association between early changes in physical dysfunction and longitudinal changes in health-related quality of life, our study contributes essential insights that can inform timely interventions and personalised treatment plans. It provides a foundation for a more patient-centred healthcare approach, emphasising individualised care based on risk profiles. Additionally, our findings offer valuable data for optimising resource allocation, healthcare planning, and patient education. This work underscores the significance of routinely assessing physical function as part of quality of life measures, potentially leading to more tailored interventions. Furthermore, our research initiates a pathway for further in-depth investigations into the mechanisms connecting early physical dysfunction to long-term well-being, offering a rich avenue for future research endeavours in the field.

## 5. Conclusions

In conclusion, to our knowledge, this is the first study to show that the degree of recovery of physical function, as assessed using HGS, is associated with long-term health outcomes and the health-related quality of life of COVID-19 survivors. This has important implications for patient management that go beyond COVID-19. The implementation of strategies to limit muscle impairment and muscle loss might minimize the risk of cardiometabolic alterations and adverse long-term outcomes not only in patients who had COVID-19, but also in those undergoing prolonged bed rest, suffering from systemic inflammatory conditions, or exposed to other catabolic stimuli. From this perspective, we recommend that HGS evaluation be integrated in the routine clinical evaluation of such patients, as it is a useful, easy-to-use, inexpensive, and reliable tool to evaluate muscle strength and physical function. Overall, our findings have the potential to improve the care of COVID-19 survivors by helping to identify those who are at risk of long-term physical impairment and who may benefit from additional support.

## Figures and Tables

**Table 1:** Patients’ demographic characteristics upon admission and during in-hospital clinical disease course.

Variables	
Age, median (IQR) years	67 (56–74.3)
Males, n (%)	144 (61.5%)
Race/Ethnicity, n (%) - White - Hispanic - Asian - Black	218 (93.2) 10 (4.2) 3 (1.3) 3 (1.3)
Active smokers, n (%)	53 (22.6%)
Arterial hypertension, n (%)	136 (58.1%)
Diabetes mellitus, n (%)	50 (21.4%)
Ischemic heart disease, n (%)	39 (16.7%)
COPD/asthma, n (%)	23 (9.8%)
Chronic kidney disease, n (%)	18 (7.7%)
Active malignancy, n (%)	7 (3%)
BMI, median (IQR) kg/m^2^	28.2 (25.6–31.2)
Length of stay, median (IQR) days	14 (9–24)
Low-flow oxygen, n (%)	138 (59%)
NIV, n (%)	62 (26.5%)
ICU, n (%)	34 (14.5%)

Continuous variables are expressed as median (25th and 75th percentiles). Categorical variables are expressed as absolute values (%). Abbreviations: COPD, Chronic Obstructive Pulmonary Disease; BMI, body mass index; NIV, non-invasive ventilation; ICU, intensive care unit.

**Table 2:** Comparison between patients with low or normal handgrip strength (HGS) at 1 month after discharge.

Variable	Low HGS (n = 82)	Normal HGS (n = 152)	*p* Value
Age, years	60.0 (54.0; 69.0)	70.0 (60.0; 77.0)	<0.001
Female sex, n (%)	24 (29.3)	66 (43.4)	0.034
Ethnicity, n (%) - White - Hispanic - Asian - Black	72 (87.8) 5 (6.1) 2 (2.4) 3 (3.7)	146 (96.1) * 5 (3.3) 1 (0.7) 0 (0)	0.031
Smoke, n (%)	20 (24.2)	33 (21.9)	0.659
BMI (baseline), kg/m^2^	28.5 (25.8; 31.4)	28.0 (25.3; 31.2)	0.395
BMI category ^†^, n (%) - Underweight - Normal weight - Overweight - Obesity	0 (0) 14 (17.3) 37 (45.7) 30 (37.0)	1 (0.6) 33 (22.0) 64 (42.7) 52 (34.7)	0.800
Arterial hypertension, n (%)	46 (56.1)	90 (59.2)	0.645
Diabetes mellitus, n (%)	22 (26.8)	28 (18.4)	0.134
Coronary artery disease, n (%)	17 (20.7)	22 (14.5)	0.220
Chronic kidney disease, n (%)	7 (8.5)	11 (7.2)	0.722
COPD/asthma, n (%)	8 (9.8)	15 (9.9)	0.978
Malignancy, n (%)	1 (1.2)	6 (3.9)	0.426
Treatment modality, n (%) - Low-flow oxygen - NIV - ICU	30 (36.6) 27 (32.9) 25 (30.5)	108 (71.1) ** 35 (23.0) 9 (5.9) **	<0.001
Length of stay, days	21.0 (10.0; 40.5)	12.0 (8.0; 20.0)	<0.001
**Follow-up: 1 month**			
Time from discharge, days	34.5 (29.0; 39.8)	34.0 (29.0; 39.0)	0.970
SBP, mmHg	130.0 (120.0; 140.0)	130.0 (120.0; 140.0)	0.163
DBP, mmHg	80.0 (70.0; 80.0)	80.0 (70.0; 80.0)	0.555
SatO_2_, %	98.0 (97.0; 99.0)	98.0 (97.0; 99.0)	0.624
Dyspnoea ^‡^, n (%)	20 (27.4)	28 (19.9)	0.286
Weight change (0–1 month)	−5.7 (−9.1; −0.6)	−3.2(−5.7; 0.0)	0.004
BMI, kg/m^2^	27.2 (25.2; 29.6)	27.6 (24.6; 30.7)	0.574
Abdominal obesity, n (%)	38 (46.3)	96 (63.2)	0.013
Handgrip strength, kg	18.6 (13.8; 25.4)	23.4 (16.6; 31.5)	<0.001
6-MWT, m	460.0 (400.0; 500.0)	460.0 (440.0; 500.0)	0.486
6-MWT, % predicted	91.0 (81.0; 96.0)	93.0 (86.6; 101.0)	0.012
**Follow-up: 3 months**			
Time from discharge, days	90.0 (90.0; 96.0)	90.0 (90.0; 93.0)	0.978
SBP, mmHg	130.0 (120.0; 135.0)	130.0 (120.0; 140.0)	0.036
DBP, mmHg	80.0 (73.8; 80.0)	80.0 (70.0; 80.0)	0.600
SatO_2_, %	98.0 (97.0; 98.0)	98.0 (98.0; 98.0)	0.360
Dyspnoea ^‡^, n (%)	13 (15.9)	16 (10.5)	0.053
BMI, kg/m^2^	27.5 (25.4; 29.9)	27.7 (24.6; 30.8)	0.923
Abdominal obesity, n (%)	45 (54.9)	98 (64.5)	0.151
Handgrip strength, kg	21.5 (14.7; 27.6)	24.2 (16.6; 33.1)	0.013
6-MWT, m	480.0 (410.0; 500.0)	480.0 (460.0; 500.0)	0.979
6-MWT, % predicted	95.7 (84.0; 102.0)	100.0 (92.9; 105.0)	0.007
**Follow-up: 6 months**			
Time from discharge, days	180.0 (179.0; 181.3)	180.0 (180.0; 188.0)	0.136
SBP, mmHg	125.0 (120.0; 135.0)	130.0 (120.0; 140.0)	0.041
DBP, mmHg	80.0 (75.0; 80.0)	80.0 (80.0; 80.0)	0.729
SatO_2_, %	98.0 (97.0; 98.0)	98.0 (98.0; 98.0)	0.189
Dyspnoea ^‡^, n (%)	10 (12.2)	14 (9.2)	0.449
BMI (1 month), kg/m^2^	27.9 (25.5; 30.8)	27.8 (24.8; 31.2)	0.824
Abdominal obesity, n (%)	50 (61.0)	103 (67.8)	0.298
Handgrip strength, kg	24.5 (15.8; 33.3)	25.0 (18.4; 32.9)	0.433
6-MWT, m	500.0 (460.0; 520.0)	480.0 (460.0; 500.0)	0.881
6-MWT, % predicted	100.0 (89.2; 105.0)	102 (96.3; 109.0)	0.003

Continuous variables are expressed as median (25th and 75th percentiles). Categorical variables are expressed as absolute values (%). Abbreviations: BMI, body mass index; DBP, diastolic blood pressure; ICU, intensive care unit; MNA-SF, mini nutritional assessment—short form; NIV, non-invasive mechanical ventilation; SatO2, peripheral oxygen saturation; SBP, systolic blood pressure; 6-MWT, 6 min walking test. Percentages were calculated using the actual number of cases. Missing: ^†^ 3; ^‡^ 20 at 1 month, 13 at 3 months, 15 at 6 months. * *p* < 0.05; ** *p* < 0.01.

**Table 3:** Comparison between patients with low handgrip strength (HGS) at 1 month after discharge whose handgrip strength improved or did not normalize at 6 months after discharge.

Variable	Stable/Worse HGS (n = 31)	Improved HGS (n = 51)	*p* Value
Age, years	56.0 (51.0; 67.0)	62.0 (56.0; 72.0)	0.094
Female sex, n (%)	11 (35.5)	13 (25.5)	0.335
Smoke, n (%)	8 (25.8)	12 (23.5)	0.816
BMI (baseline), kg/m^2^	28.3 (25.6 (31.9)	28.5 (25.8; 31.3)	0.977
BMI category ^†^, n (%) - Underweight - Normal weight - Overweight - Obesity	- 5 (16.1) 13 (41.9) 12 (38.7)	- 9 (17.6) 24 (47.1) 18 (35.3)	0.543
Arterial hypertension, n (%)	19 (61.3)	27 (52.9)	0.460
Diabetes mellitus, n (%)	9 (29.0)	13 (25.5)	0.726
Coronary artery disease, n (%)	9 (29.0)	8 (15.7)	0.148
Chronic kidney disease, n (%)	3 (9.7)	4 (7.8)	1.000
COPD/asthma, n (%)	5 (16.1)	3 (5.9)	0.129
Malignancy, n (%)	0 (0)	1 (2.0)	1.000
Treatment modality, n (%) - Low-flow oxygen - NIV - ICU	11 (35.5) 9 (29.0) 11 (35.5)	19 (37.3) 18 (35.3) 14 (27.5)	0.721
Length of stay, days	19.0 (9.0; 43.0)	21.0 (10.8; 39.8)	0.596
**Follow-up: 1 month**			
Time from discharge, days	35.0 (29.0; 38.3)	33.5 (29.0; 40.0)	0.964
SBP, mmHg	130.0 (110.0; 140.0)	130.0 (120.0; 135.0)	0.399
DBP, mmHg	80.0 (70.0; 85.0)	80.0 (70.0, 80.0)	0.941
SatO_2_, %	98.0 (97.0; 99.0)	98.0 (97.0; 98.0)	0.477
Dyspnoea ^‡^, n (%)	11 (35.5)	9 (17.6)	0.140
Weight change 0–1 months, %	−4.7 (−9.8; 0.0)	−5.9 (−8.7; −1.6)	0.784
MNA-SF			0.362
No malnutrition	3 (10.0)	4 (7.8)	
Risk of malnutrition	16 (53.3)	20 (39.2)	
Malnutrition	11 (36.7)	27 (52.9)	
BMI (1 month), kg/m^2^	27.3 (25.2; 29.0)	27.1 (25.0; 30.5)	0.800
Waist circumference (cm)	95.0 (88.0; 103.0)	97.0 (90.0; 108.0)	0.464
Abdominal obesity, n (%)	17 (54.8)	21 (41.2)	0.229
Capillary blood glucose, mg/dL	108.0 (100.0; 120.0)	114.0 (101.0; 149.0)	0.176
Handgrip strength, kg	15.8 (12.6; 19.6)	22.2 (13.9; 26.0)	0.006
6-MWT, m	460.0 (320.0; 500.0)	460.0 (420.0; 500.0)	0.265
6-MWT, % predicted	87.0 (73.0; 93.3)	91.5 (84.3; 97.8)	0.006
**Follow-up: 3 months**			
Time from discharge, days	90.0 (90.0; 96.0)	90.0 (90.0; 97.0)	0.900
SBP, mmHg	130.0 (120.0; 135.0)	130.0 (115.0; 130.0)	0.506
DBP, mmHg	80.0 (70.0; 80.0)	80.0 (75.0; 80.0)	0.649
SatO_2_, %	98.0 (97.0; 98.0)	98.0 (97.0; 98.0)	0.882
Dyspnoea ^‡^, n (%)	9 (29.0)	4 (7.8)	0.022
BMI (1 month), kg/m^2^	27.5 (25.0; 29.6)	27.4 (25.5; 30.2)	0.992
Waist circumference (cm)	95.0 (90.0; 110.0)	97.0 (90.0; 108.0)	0.989
Abdominal obesity, n (%)	21 (67.7)	24 (47.1)	0.068
Capillary blood glucose, mg/dL	122.0 (106.0; 145.0)	116.0 (103.0; 147.0)	0.681
HGS, kg	17.0 (12.4; 22.7)	25.0 (17.3; 29.1)	0.001
6-MWT, m	470.0 (355.0; 505.0)	500.0 (440.0; 500.0)	0.205
6-MWT, % predicted	87.0 (73.8; 95.7)	98.8 (92.0; 103.0)	<0.001
**Follow-up: 6 months**			
Time from discharge, days	180.0 (179.0; 181.0)	180.0 (179.0; 188.0)	0.193
SBP, mmHg	125.0 (120.0; 130.0)	125.0 (120.0; 135.0)	0.301
DBP, mmHg	80.0 (70.0; 80.0)	80.0 (80.0; 80.0)	0.146
SatO_2_, %	98.0 (97.0; 98.0)	98.0 (98.0; 98.0)	0.408
Dyspnoea ^‡^, n (%)	9 (29.0)	1 (2.0)	<0.001
BMI (1 month), kg/m^2^	27.7 (25.5; 30.8)	28.1 (25.6; 30.7)	0.916
Waist circumference (cm)	100.0 (92.0; 110)	101.0 (92.0; 108.0)	0.912
Abdominal obesity, n (%)	21 (67.7)	29 (56.9)	0.327
Capillary blood glucose, mg/dL	123.0 (109.0; 163.0)	116.0 (101.0; 150.0)	0.341
Handgrip strength, kg	19.4 (14.4; 24.3)	31.2 (21.3; 35.9)	<0.001
6-MWT, m	480.0 (430.0; 505.0)	500.0 (460.0; 520.0)	0.355
6-MWT, % predicted	93.3 (78.3; 101.0)	101.0 (95.0; 107.0)	<0.001

Continuous variables are expressed as median (25th and 75th percentiles). Categorical variables are expressed as absolute values (%). Abbreviations: BMI, body mass index; DBP, diastolic blood pressure; ICU, intensive care unit; MNA-SF, mini nutritional assessment—short form; NIV, non-invasive mechanical ventilation; SatO2, peripheral oxygen saturation; SBP, systolic blood pressure, 6-MWT, 6 min walking test. Percentages are calculated on the actual number of cases. Missing: ^†^ 1; ^‡^ 9 (1 month), 8 (3 months), 7 (6 months).

**Table 4:** Binomial univariable and multivariable logistic regression for HGS improvement (low to normal) from 1 to 6 months after discharge in patients with low HGS at the 1-month follow-up visit (n = 82).

Variable	Univariable		Multivariable	
	Odds Ratio (95% C.I.)	*p* Value	Odds Ratio (95% C.I.)	*p* Value
Age	1.03 (0.99; 1.07)	0.145		
Sex (female)	1.61 (0.61; 4.23)	0.337		
Race (white)	4.67 (1.01; 19.67)	0.036	4.37 (0.97; 19.70)	0.055
BMI (1 M)	0.97 (0.89; 1.06)	0.476		
Abdominal obesity (1 M)	1.74 (0.71; 4.27)	0.231		
Arterial hypertension	0.711 (0.287; 1.76)	0.461		
Diabetes mellitus	0.84 (0.31; 2.27)	0.726		
Coronary artery disease	0.46 (0.15; 1.34)	0.154		
Chronic kidney disease	0.79 (0.67; 3.81)	0.774		
COPD/asthma	0.33 (0.07; 1.47)	0.144		
ICU	0.69 (0.26; 1.80)	0.445		
LoS	1.00 (0.98; 1.02)	0.930		
Handgrip strength 1 M	1.12 (1.03; 1.21)	0.006	1.11 (1.03; 1.20)	0.008

Abbreviations: BMI, body mass index; COPD chronic obstructive pulmonary disease; ICU intensive care unit; LoS, length of stay; 1 M, one month.

## Data Availability

The data presented in this study are available on request from the corresponding author.
